# Evaluation of the relationship between plasma lipids and abdominal aortic aneurysm: A Mendelian randomization study

**DOI:** 10.1371/journal.pone.0195719

**Published:** 2018-04-12

**Authors:** Lu-Chen Weng, Nicholas S. Roetker, Pamela L. Lutsey, Alvaro Alonso, Weihua Guan, James S. Pankow, Aaron R. Folsom, Lyn M. Steffen, Nathan Pankratz, Weihong Tang

**Affiliations:** 1 Division of Epidemiology and Community Health, School of Public Health, University of Minnesota, Minneapolis, Minnesota, United States of America; 2 Division of Biostatistics, School of Public Health, University of Minnesota, Minneapolis, Minnesota, United States of America; 3 Department of Laboratory Medicine and Pathology, University of Minnesota, Minneapolis, Minnesota, United States of America; Tulane University School of Public Health and Tropical Medicine, UNITED STATES

## Abstract

Studies have reported that higher circulating levels of total cholesterol (TC), low-density lipoprotein (LDL) cholesterol and lower of high-density lipoprotein (HDL) cholesterol may be associated with increased risk of abdominal aortic aneurysm (AAA). Whether dyslipidemia causes AAA is still unclear and is potentially testable using a Mendelian randomization (MR) approach. We investigated the associations between blood lipids and AAA using two-sample MR analysis with SNP-lipids association estimates from a published genome-wide association study of blood lipids (n = 188,577) and SNP-AAA association estimates from European Americans (EAs) of the Atherosclerosis Risk in Communities (ARIC) study (n = 8,793). We used inverse variance weighted (IVW) MR as the primary method and MR-Egger regression and weighted median MR estimation as sensitivity analyses. Over a median of 22.7 years of follow-up, 338 of 8,793 ARIC participants experienced incident clinical AAA. Using the IVW method, we observed positive associations of plasma LDL cholesterol and TC with the risk of AAA (odds ratio (OR) = 1.55, *P* = 0.02 for LDL cholesterol and OR = 1.61, *P* = 0.01 for TC per 1 standard deviation of lipid increment). Using the MR-Egger regression and weighted median methods, we were able to validate the association of AAA risk with TC, although the associations were less consistent for LDL cholesterol due to wider confidence intervals. Triglycerides and HDL cholesterol were not associated with AAA in any of the MR methods. Assuming instrumental variable assumptions are satisfied, our finding suggests that higher plasma TC and LDL cholesterol are causally associated with the increased risk of AAA in EAs.

## Introduction

Abdominal aortic aneurysm (AAA) affects about 1–2% of women and 4%-8% of men over 65 years old based on screening studies.[1] AAA is usually asymptomatic and the mortality rate after its rupture may exceed 90%.[2] Identification of risk factors for AAA are important for primary prevention of AAA as well as decreasing AAA-related mortality.

Several risk factors for AAA have been reported, including greater age[3, 4], male sex[4, 5], white race[5–8], cigarette smoking, and genetic factors.[9] A few prospective studies tested the associations between lipids and AAA risk, and they reported an inverse relationship between high-density lipoprotein (HDL) and AAA and positive relationships between total cholesterol (TC), low-density lipoprotein (LDL), triglycerides and AAA.[10–14]

The Mendelian randomization approach is a method to estimate the causal effect of an exposure on a disease outcome by using genetic variant(s) as instrumental variable(s) (IVs) of the exposure. Based on several assumptions, the causal effect of the exposure on the outcome can be estimated via the IV (genetic variant), regardless of the presence of confounding variables between the exposure and outcome.[15] The three main assumptions for Mendelian randomization include that the IV: (i) is associated with the exposure; (ii) is not associated with any confounder of the exposure—outcome association; and (iii) is conditionally independent of the outcome, given the exposure and confounders. A fourth IV assumption (monotonicity) is also required in order to identify a causal effect. We aimed to conduct a Mendelian randomization study to investigate a potential causal link between plasma lipids and AAA in the European Americans (EAs) of the Atherosclerosis Risk in Communities (ARIC) Study, using lipid-related single nucleotide polymorphisms (SNPs) as IVs.

## Materials and methods

### Study population

The ARIC study[16] enrolled a total of 15,792 participants aged 45–64 to a baseline exam in 1987–1989 from four U.S. communities, namely Forsyth County, North Carolina; Jackson, Mississippi; suburbs of Minneapolis, Minnesota; and Washington County, Maryland. Trained interviewers collected demographic, medical, and lifestyle information at baseline and four follow-up exams. All medications and supplements taken in the two weeks prior to the baseline exam were also recorded, and medication names were transcribed and coded. Each participant provided informed consent, and the Institutional Review Board at the University of Minnesota, Johns Hopkins University, Wake Forest University, University of North Carolina at Chapel Hill, Baylor College of Medicine, University of Texas Health Sciences Center at Houston, and University of Mississippi Medical Center approved the protocol.

### Plasma lipid measurements

Fasting blood samples were collected at the baseline exam and stored at −70°C for a few weeks prior to lipid measurement. The ARIC Central Lipid Laboratory measured plasma cholesterol and triglycerides using a Cobas-Bio centrifugal analyzer (Roche Diagnostics, Montclair, NJ) with enzymatic kits (Boehringer Mannheim Diagnostics, Indianapolis, IN).[17, 18] HDL cholesterol was estimated by the method of Warnick et al,[19] and LDL cholesterol was calculated using the Friedewald formula.[20] If the level of triglycerides was over 400 mg/dL, then LDL cholesterol was not determined. The coefficients of variation within the laboratory for TC, triglycerides, HDL cholesterol, and LDL cholesterol were 2.5%, 2.7%, 3.7%, and 5.2%, respectively.[21]

### SNP selection

We selected the SNP IVs for each lipid fraction based on the results of the Global Lipids Genetics Consortium (GLGC) genome-wide association study[22] with MR-Base (www.mrbase.org), [23] using the default thresholds for statistical significance (P < 5 x 10^−8^) and pruning SNPs for linkage disequilibrium (r^2^ = 0.001). A total of 78, 85, 53, and 85 SNPs were selected as the IVs for LDL cholesterol, HDL cholesterol, triglycerides, and TC, respectively (see S1 Table for additional information about these SNPs).

### Genotyping and imputation

The Affymetrix Genome-Wide Human SNP Array 6.0 (Affymetrix, Santa Clara, CA, USA) was used to genotype SNPs in ARIC; imputation was conducted with the 1000 genome phase I version 3 reference panel. Detailed information on GWAS genotyping, quality control and imputation can be found elsewhere.[24] Imputation quality for the SNPs included in this study was good (quality score = 0.84–1.0 with a mean of 0.99). Additionally, 10 principal components of population stratification based on the GWAS data were generated by EIGENSTRAT[25] to reflect the population structure of the ARIC participants.

### Outcome measurement

Incident, clinical AAA was defined using ICD codes from hospitalization records and death records from baseline through 2011, and the Medicare records from Centers for Medicare and Medicaid Services (CMS) for 1991–2011. In ARIC’s annual phone interview, each ARIC participant was asked about all hospitalizations and these records were sought. Additionally, participant identifiers were linked to Medicare claims to identify any missing AAA cases. Clinical AAA cases were defined based on one hospital discharge diagnosis or 2 outpatient diagnoses that occurred at least one week apart (ICD-9-CM: 441.3, or 441.4), procedure codes (38.44 or 39.71), or underlying cause of death codes (ICD-9: 441.3 or 441.4 or ICD-10: I71.3, or I71.4). Other diagnostic codes that indicated a probable diagnosis of AAA were investigated case-by-case to rule out AAA diagnosis. People with known AAA surgery at baseline (n = 11) or uncertain AAA during follow-up (n = 30) were excluded. Details on AAA ascertainment are described elsewhere.[14]

### Statistical analysis

We removed individuals from the analysis if they were non-white (n = 4,308), missing genotype data (n = 1,980), taking cholesterol lowering medication (n = 319), not fasting for at least 8 hours (n = 206), or missing any lipid fraction measurements (n = 145).

We estimated odds ratios (OR) and 95% confidence intervals (CI) for AAA per 1 standard deviation increment in each of the lipid fractions using two-sample Mendelian randomization techniques, with the inverse variance weighted (IVW) approach as the primary method and MR-Egger regression and weighted median estimation approaches as sensitivity analyses to validate the results.[26, 27] In the primary analysis, we used the ratio estimator^5^ to combine the summary estimates and standard errors of the SNP—lipid fraction associations from the GLGC (median sample size: 177,765) with the summary estimates and standard errors of the SNP—AAA associations (adjusted for age, sex, and study center) from ARIC. We calculated the I^2^ index and *Q* test^6^ from the IVW analysis to check for evidence of heterogeneity among the IV estimates.

There might be bias in the estimates obtained in the IVW approach if any of the SNPs do not satisfy the IV assumptions. In particular, the assumption (iii) may be violated due to pleiotropy (i.e., if a SNP is associated with AAA risk through a pathway that does not involve blood lipids). Therefore, we also conducted sensitivity analyses using two other MR methods (MR-Egger regression and weighted median estimation) that allow for some relaxation of the IV assumptions. MR-Egger regression allows all IVs to violate the assumption (iii) as long as they meet a weaker assumption that any pleiotropic effects of the IVs are uncorrelated with the strength of their associations with the exposure.[26] Additionally, the intercept term from MR-Egger regression serves as a check for evidence of directional pleiotropy.[26] Weighted median estimation requires that no more than 50% of the weight contributed by the genetic variants violates the IV assumptions (ii) and (iii).[27] All MR analyses were conducted in Stata (version 12.1) using the *mrrobust* package.

We also conducted two additional sensitivity analyses. In the first one, the SNP—AAA associations from ARIC were additionally adjusted for the first five principal components of ancestry to account for population stratification. The second sensitivity analysis used ARIC as the data source for both the SNP—lipid fraction associations and SNP—AAA associations, with both sets of summary estimates adjusted for age, sex, and study center. Because ARIC comprised approximately 4.4% of the samples used in the GLGC genome-wide association study, there is a possibility of weak instrument bias in two-sample Mendelian randomization since the summary estimates were based on overlapping samples. Likewise, the second sensitivity analysis is even more prone to this bias given the 100% overlap of samples used to derive the summary estimates. As such, we estimated the strength of the IVs using the F statistic, the expected bias due to sample overlap under the null hypothesis, and the expected type I error rate.[28]

## Results

A total of 8,793 EAs had measurements for plasma lipids, SNP dosages, and follow-up for AAA and thus were included in our analysis. Baseline characteristics for those who developed clinical AAA (n = 388) and those who did not (n = 8,405) after a median of 22.7 years of follow-up are shown in Table 1. Mean values for age, height, white blood count, alcohol intake, fibrinogen, LDL cholesterol, triglycerides, and TC were higher in those who developed clinical AAA than the remaining participants. The percentage of men, current smokers, and those with peripheral artery disease and hypertension were higher in incident clinical AAA cases as well. In contrast, mean HDL cholesterol levels and the percentage with diabetes were lower in clinical AAA cases.

**Table 1:** Baseline characteristics [means (SD) or %] in 1987–89 for those who did or did not develop incident abdominal aortic aneurysm, ARIC European Americans.

Characteristic	No AAA (N = 8,405)	Developed AAA (N = 388)
Age, years	54 (6)	57 (5)
Female, %	55	25
Height, cm	168 (9)	173 (8)
Fibrinogen, mg/dL	295 (60)	318 (65)
White blood count, x1,000 cell/mm^3^	6.2 (2.0)	7.0 (2.0)
Alcohol intake, g/week	45 (91)	71 (126)
Current smoker, %	23	51
Hypertension, %	25	35
Diabetes, %	7.7	4.9
Peripheral arterial disease, %	1.8	4.8
LDL cholesterol, mg/dL	136 (37)	149 (35)
HDL cholesterol, mg/dL	51 (17)	44 (14)
Triglycerides, mg/dL	128 (65)	143 (71)
TC, mg/dL	214 (40)	222 (37)

SD: standard deviation; LDL cholesterol: low-density lipoprotein cholesterol; HDL cholesterol: high-density lipoprotein cholesterol; TC: total cholesterol

Table 2 shows the strength of the SNP IVs in the GLGC and ARIC and their susceptibility to weak instrument bias. The SNP IVs explained more of the variation in LDL cholesterol and TC than in the other lipid fractions. In comparing the data sources, the SNPs generally explained slightly more variation of the lipid fractions in ARIC than in the GLGC, although F-statistics were much larger for the latter study because of its larger sample size (N = 188,577 in GLGC vs N = 8,793 in ARIC). The bias resulting from weak instruments for overlapped two-sample MR analysis would be limited using the GLGC data, and the expected bias under the null hypothesis and type I error rates were generally negligible or low when using ARIC data with 100% sample overlap (Table 2).

**Table 2:** Estimated strength of SNP IVs and susceptibility to weak instrument bias using the two-sample Mendelian randomization approach for estimating the association between lipids and AAA risk.

Exposure	# of SNP IVs	SNP-lipid summary estimate source	r^2^*	F^†^	Beta^‡^ (P-value)	Sample overlap	Bias under null^†^	Type I error rate^†^
LDL cholesterol	78	GLGC	0.071	184.7		4.4%	0.0001	0.050
		ARIC	0.087	10.7	0.2391 (<0.001)	100%	0.0223	0.052
HDL cholesterol	85	GLGC	0.050	116.7		4.4%	<0.0001	0.050
		ARIC	0.057	6.2	-0.1224 (0.13)	100%	-0.0197	0.051
Triglycerides	53	GLGC	0.044	163.7		4.4%	<0.0001	0.050
		ARIC	0.064	11.2	0.0618 (0.48)	100%	0.0055	0.050
TC	85	GLGC	0.066	156.7		4.4%	0.0001	0.050
		ARIC	0.089	10.0	0.2372 (<0.001)	100%	0.0237	0.052

LDL cholesterol: low-density lipoprotein cholesterol; HDL cholesterol: high-density lipoprotein cholesterol; TC: total cholesterol

*Percentage of variance in the lipids explained by the SNPs

^†^The F statistic, bias under the null, and the type I error rate values were estimated using equations from Burgess et al.[28]

^‡^In ARIC, the log-odds ratio for AAA per 1 standard deviation (SD) increment in the lipid fraction using traditional logistic regression, adjusted for age, sex, study center, smoking status, height, white blood count, fibrinogen, hypertension, diabetes, peripheral arterial disease, and the other lipid fractions (for LDL cholesterol, HDL cholesterol, and triglycerides models only)

SDs from GLGC (LDL cholesterol: 38.7 mg/dL; HDL cholesterol: 15.5 mg/dL; triglycerides: 90.7 mg/dL; total cholesterol: 41.8 mg/dL)

Table 3 shows the two-sample Mendelian randomization estimates of ORs (95% CIs) for the association of AAA with lipids, with the GLGC and ARIC as the data sources for the SNP—lipid and SNP—AAA associations, respectively. Higher TC was associated with a greater risk of AAA (OR 1.48, 95% CI: 1.02, 2.16 per 41.8 mg/dL increment) as estimated by the IVW method; ORs estimated using the MR-Egger and weighted median methods were similar in magnitude (OR: 2.04 and 1.66) with wider confidence intervals (p = 0.03 and 0.09, respectively). Similarly, each 38.7 mg/dL increment in LDL cholesterol was associated with 1.55 (95% CI: 1.08, 2.22) times greater odds of AAA based on the IVW methods. The MR-Egger regression and weighted median estimates were similar (1.43 and 1.59) but with wider confidence intervals (p = 0.21 and 0.09, respectively). None of the IV methods showed evidence of associations for HDL cholesterol or triglycerides with the risk of AAA.

**Table 3:** (Primary analysis). Two-sample Mendelian randomization results for the odds of AAA per 1 SD increment in lipid fraction measures. SNP—lipid associations are from the GLGC and SNP—AAA associations (adjusted for age, sex, and center) are from ARIC.

Exposure	Method	OR	95% CI	*P*	I^2^ (95% CI)	*P* for Q test	MR-Egger intercept (95% CI)	*P*
LDL cholesterol	MR-IVW	1.55	(1.08, 2.22)	0.02	5% (0, 28%)	0.35		
	MR-Egger	1.43	(0.81, 2.49)	0.21			0.01 (-0.03, 0.04)	0.70
	MR-Weighted median	1.59	(0.93, 2.73)	0.09				
HDL cholesterol	MR-IVW	0.68	(0.42, 1.12)	0.13	29% (7, 46%)	0.007		
	MR-Egger	0.56	(0.22, 1.42)	0.22			0.01 (-0.03, 0.05)	0.62
	MR-Weighted median	0.62	(0.32, 1.21)	0.16				
Triglycerides	MR-IVW	1.08	(0.64, 1.80)	0.77	17% (0, 42%)	0.15		
	MR-Egger	1.91	(0.85, 4.32)	0.12			-0.03 (-0.07, 0.004)	0.08
	MR-Weighted median	1.09	(0.50, 2.39)	0.83				
TC	MR-IVW	1.48	(1.02, 2.16)	0.04	5% (0, 27%)	0.36		
	MR-Egger	2.04	(1.07, 3.87)	0.03			-0.02 (-0.05, 0.01)	0.23
	MR-Weighted median	1.66	(0.92, 3.00)	0.09				

SD: standard deviation; LDL cholesterol: low-density lipoprotein cholesterol; HDL cholesterol: high-density lipoprotein cholesterol; TC: total cholesterol; IVW: inverse variance weighted

SDs from GLGC (LDL cholesterol: 38.7 mg/dL; HDL cholesterol: 15.5 mg/dL; triglycerides: 90.7 mg/dL; total cholesterol: 41.8 mg/dL)

There was no evidence of heterogeneity in the effect estimates used in the IVW analysis across the individual SNPs for LDL cholesterol and TC (I^2^ = 5%, *P* ≥ 0.35) but possibly modest heterogeneity for HDL cholesterol (I^2^ = 29%, *P* = 0.007; Table 3). The intercept estimates in the MR-Egger models for the four lipid fractions indicated no evidence for directional pleiotropy (*P* ≥ 0.08, Table 3). Sensitivity analyses that additionally adjusted for the first five principal components of ancestry (S2 Table) or used ARIC (instead of the GLGC) as the data source for the SNP—lipid summary estimates (S3 Table) were overall very consistent with the primary results shown in Table 3.

## Discussion

Using the IVW method for two-sample MR, we demonstrated positive associations of LDL cholesterol and TC with the incidence of clinical AAA in EAs from the prospective, population-based ARIC study. The association between TC and AAA risk was supported by MR-Egger and median weighted methods with similar effect sizes and p-values, while the association between LDL cholesterol and AAA risk was less consistent due to the wider confidence intervals in both methods. No significant association was observed between HDL cholesterol or triglycerides and AAA.

A pleiotropic effect is a major concern related to the third assumption of Mendelian randomization.[15] An instrumental variable that has an effect on multiple risk factors simultaneously could distort or bias the estimates in our Mendelian randomization study. Using lipid SNPs as the instrument in Mendelian randomization studies of cardiovascular disease has been recognized as a challenge[29] because of the complex nature of the genetic influences for lipids. These complexities include (i) the close correlation among plasma lipid fractions, (ii) many identified SNPs being novel, with unclear function in lipid pathways and (iii) many of the SNPs being associated with multiple lipid fractions. Instead of removing variants related to multiple lipids, which likely reduces statistical power, we performed MR-egger method and demonstrated that the bias resulting from directional pleiotropic effects should be minimal (P > 0.05 for MR-Egger intercepts differing from zero).

In general, weak instruments may bias the estimated association between exposures and health outcomes.[15] In the current study, we included multiple genetic variants as IVs for plasma lipids and observed that our IVs as a whole had high F-statistics and explained a significant percentage of variance in lipid levels. This suggests that bias resulting from weak instruments is unlikely. Furthermore, given the relatively small amount of overlap between samples in our primary analysis (SNP—lipid associations from GLGC and SNP—AAA associations from ARIC, with 4.4% overlap), weak instruments would lead to bias towards the null.[30] In addition, a simulation study conducted by one of our coauthors (Guan et al.) showed that the bias is ignorable when the overlap is less than 20% (unpublished data). Our sensitivity analysis using estimates derived from ARIC for both SNP-lipids and SNP-AAA associations showed concordant results to the primary analysis. Bias due to weak instruments in a one-sample setting leads to bias towards the confounded observational association,[30] although there was also little concern of weak instruments as indicated in our sensitivity analysis. Significant associations between serum TC and AAA risk were previously observed in several perspective cohort studies, with higher serum TC associated with greater risk of AAA.[10–14] Provided the SNPs used in this analysis are valid instruments, our findings suggest a causally positive relation between TC and AAA, and the similar results from MR-egger and median weighted methods support this interpretation. Our results using the IVW method also showed that the LDL cholesterol could be causally related to AAA risk, and the results from the other two methods were very similar in the association estimates, although were less precise as reflected by the wider confidence interval. This is not surprising, because the IVW MR method is generally known to have better precision than MR-Egger regression and weighted median estimation.[27] The similar patterns of associations of TC and LDL cholesterol with AAA risk may imply that the causal effect from TC to AAA risk could be largely attributable to the effect from LDL cholesterol.

Mendelian randomization analysis did not support a causal association of HDL cholesterol or triglycerides with clinical AAA incidence in our sample. One explanation is that the genetic variants for HDL cholesterol and triglycerides only explained a small proportion of the total variance in these lipid levels (r^2^ = 5% for HDL cholesterol and r^2^ = 4.4% for triglycerides), which may have reduced our ability to detect a true association between these two lipids and AAA risk. Another interpretation is that both lipids are not causally related to AAA risk.

The biological mechanism underlying the association between TC and AAA is unclear. Animal studies showed that oxidized lipoproteins promote recruitment of inflammatory cells into the elastic media and adventitia of the aortic wall.[31] In vitro studies reported an inverse association between statin use and the activity of matrix metalloproteinase-9, a biomarker for AAA development.[32] Additionally, clinical trials have shown that statin use slows AAA growth.[32] These findings may potentially support a causal pathway between TC or LDL cholesterol and AAA risk; however, statins may prevent AAA by reducing inflammation as well.[32]

Recently, a large meta-analysis MR study between AAA and lipids reported significant associations of AAA with LDL-C, HDL-C, and triglycerides.[33] The strength of association for LDL-C and HDL-C in that study was similar to ours, while that for triglycerides was stronger. Differences in population characteristics might contribute to the heterogeneity in the strength of association for triglycerides. A prior research from Malmö Preventive Study showed increased blood triglycerides and total cholesterol in 126 male subjects later developing large AAA compared to 126 male controls after a median follow-up of 21 years, and the association between triglycerides and AAA was no longer significant after adjusting for total cholesterol and other risk factors.[12] It is noted that plasma levels of triglycerides were not significantly associated with AAA after adjustment for other cardiovascular risk factors in the longitudinal ARIC study as well.[14] The lack of statistical significance for HDL-C in our MR study was likely due to limited power. Compared to the published meta-analysis which pooled data primarily from case-control studies, our study analyzed AAAs ascertained in a prospective cohort. The significant replication of the MR association between AAA and LDL-C in our prospective study as well as the addition of total cholesterol-AAA association further strengthen the data to support a role for these lipid fractions in the etiology of AAA.

Besides the limitations related to the assumptions of Mendelian randomization analysis, there were a few other drawbacks of our study. First, the associations of genetic variants with blood lipid levels were mainly identified in individuals of European descent, and this study included only EAs. Therefore, our findings may not be generalizable to other racial/ethnic groups. Second, we used ICD codes to identify both symptomatic and asymptomatic clinical AAAs from 1991–2011. Certainly, other asymptomatic AAAs could have been present in the sample and missed without additional ultrasound screening. In our analysis, we did not include AAA cases (N = 56) identified by an ultrasound scan in ARIC visit 5 (2011–2013) occurring after our follow-up for clinical AAAs. We excluded these ultrasound AAAs because they were identified in the healthier surviving cohort and pooling them with clinical AAAs might cause bias in the risk factor associations. On the other hand, a separate analysis of the ultrasound-detected AAAs is underpowered.

## Conclusions

Using the MR approaches, we identified a significant association between AAA and TC and replicated the previously reported association for LDL-C in EAs. Our data, together with the previously published meta-analysis study, support the hypothesis that TC and LDL-C play a role in the pathogenesis of AAA in EAs. Future studies in populations of different ancestries may be required to evaluate the generalizability of these findings to different ethnic populations.

## Supporting information

S1 Table List of SNPs selected as the instrumental variables for LDL cholesterol, HDL cholesterol, triglycerides, and total cholesterol. (XLSX)

S2 Table (Sensitivity analysis). **Two-sample Mendelian randomization results for the odds of AAA per 1 standard deviation increment in lipid fraction measures.** SNP—lipid associations are from the GLGC and SNP—AAA associations (adjusted for age, sex, center, and PC1–PC5) are from ARIC.
(DOCX)

S3 Table (Sensitivity analysis). **Two-sample Mendelian randomization results for the odds of AAA per 1 standard deviation increment in lipid fraction measures.** SNP—lipid associations and SNP—AAA associations are both from ARIC and are adjusted for age, sex, and center.
(DOCX)
