# Supplementary material for: Evaluation of the relationship between plasma lipids and abdominal aortic aneurysm: A Mendelian randomization study
Source: PLoS One. 2018 Apr 12;13(4):e0195719. doi: 10.1371/journal.pone.0195719 (PMC5896990; doi:10.1371/journal.pone.0195719)
Supplement: S2 Table — Two-sample Mendelian randomization results for the odds of AAA per 1 standard deviation increment in lipid fraction measures. SNP—lipid associations are from the GLGC and SNP—AAA associations (adjusted for age, sex, center, and PC1–PC5) are from ARIC. (DOCX) [file pone.0195719.s002.docx]

**S2 Table** **(Sensitivity analysis). Two-sample Mendelian randomization results for the odds of AAA per 1 standard deviation increment in lipid fraction measures.** SNP—lipid associations are from the GLGC and SNP—AAA associations (adjusted for age, sex, center, and PC1–PC5) are from ARIC.

| **Exposure** | **Method** |  | **OR** | **95% CI** | ***P*** |  | **I^2^ (95% CI)** | ***P* for Q test** |  | **MR-Egger intercept (95% CI)** | ***P*** |
| --- | --- | --- | --- | --- | --- | --- | --- | --- | --- | --- | --- |
| LDL cholesterol | MR-IVW |  | 1.59 | (1.10, 2.28) | 0.01 |  | 5% (0, 29%) | 0.34 |  |  |  |
|  | MR-Egger |  | 1.42 | (0.81, 2.48) | 0.22 |  |  |  |  | 0.01 (-0.02, 0.04) | 0.61 |
|  | MR-Weighted median |  | 1.67 | (0.96, 2.90) | 0.07 |  |  |  |  |  |  |
| HDL cholesterol | MR-IVW |  | 0.68 | (0.41, 1.11) | 0.13 |  | 30% (7, 46%) | 0.007 |  |  |  |
|  | MR-Egger |  | 0.57 | (0.23, 1.46) | 0.24 |  |  |  |  | 0.01 (-0.03, 0.05) | 0.68 |
|  | MR-Weighted median |  | 0.55 | (0.28, 1.08) | 0.08 |  |  |  |  |  |  |
| Triglycerides | MR-IVW |  | 1.13 | (0.67, 1.91) | 0.64 |  | 19% (0, 43%) | 0.12 |  |  |  |
|  | MR-Egger |  | 2.07 | (0.91, 4.73) | 0.08 |  |  |  |  | -0.04 (-0.08, 0.003) | 0.07 |
|  | MR-Weighted median |  | 1.16 | (0.55, 2.45) | 0.70 |  |  |  |  |  |  |
| TC | MR-IVW |  | 1.50 | (1.02, 2.19) | 0.04 |  | 6% (0, 28%) | 0.32 |  |  |  |
|  | MR-Egger |  | 2.08 | (1.09, 3.98) | 0.03 |  |  |  |  | -0.02 (-0.06, 0.01) | 0.22 |
|  | MR-Weighted median |  | 1.66 | (0.91, 3.02) | 0.10 |  |  |  |  |  |  |

PC, principal component of ancestry; LDL cholesterol: low-density lipoprotein cholesterol; HDL cholesterol: high-density lipoprotein cholesterol; TC: total cholesterol; IVW: inverse variance weighted

Standard deviations (SDs) from GLGC (LDL cholesterol: 38.7 mg/dL; HDL cholesterol: 15.5 mg/dL; triglycerides: 90.7 mg/dL; TC: 41.8 mg/dL)
