# Supplementary material for: Evaluation of the relationship between plasma lipids and abdominal aortic aneurysm: A Mendelian randomization study
Source: PLoS One. 2018 Apr 12;13(4):e0195719. doi: 10.1371/journal.pone.0195719 (PMC5896990; doi:10.1371/journal.pone.0195719)
Supplement: S3 Table — Two-sample Mendelian randomization results for the odds of AAA per 1 standard deviation increment in lipid fraction measures. SNP—lipid associations and SNP—AAA associations are both from ARIC and are adjusted for age, sex, and center. (DOCX) [file pone.0195719.s003.docx]

**S3 Table** **(Sensitivity analysis). Two-sample Mendelian randomization results for the odds of AAA per 1 standard deviation increment in lipid fraction measures.** SNP—lipid associations and SNP—AAA associations are both from ARIC and are adjusted for age, sex, and center.

| **Exposure** | **Method** |  | **OR** | **95% CI** | ***P*** |  | **I^2^ (95% CI)** | ***P* for Q test** |  | **MR-Egger intercept (95% CI)** | ***P*** |
| --- | --- | --- | --- | --- | --- | --- | --- | --- | --- | --- | --- |
| LDL cholesterol | MR-IVW |  | 1.55 | (1.06, 2.26) | 0.02 |  | 6% (0, 29%) | 0.33 |  |  |  |
|  | MR-Egger |  | 1.50 | (0.83, 2.69) | 0.18 |  |  |  |  | 0.00 (-0.03, 0.04) | 0.89 |
|  | MR-Weighted median |  | 1.82 | (1.01, 3.29) | 0.05 |  |  |  |  |  |  |
| HDL cholesterol | MR-IVW |  | 0.70 | (0.43, 1.14) | 0.15 |  | 29% (7, 46%) | 0.007 |  |  |  |
|  | MR-Egger |  | 0.55 | (0.24, 1.24) | 0.15 |  |  |  |  | 0.01 (-0.02, 0.05) | 0.46 |
|  | MR-Weighted median |  | 0.76 | (0.39, 1.50) | 0.43 |  |  |  |  |  |  |
| Triglycerides | MR-IVW |  | 1.40 | (0.76, 2.58) | 0.29 |  | 15% (0, 40%) | 0.17 |  |  |  |
|  | MR-Egger |  | 2.61 | (1.05, 6.49) | 0.04 |  |  |  |  | -0.03 (-0.07, 0.003) | 0.07 |
|  | MR-Weighted median |  | 2.09 | (0.86, 5.09) | 0.11 |  |  |  |  |  |  |
| TC | MR-IVW |  | 1.61 | (1.11, 2.33) | 0.01 |  | 2% (0, 23%) | 0.42 |  |  |  |
|  | MR-Egger |  | 1.78 | (1.00, 3.14) | 0.05 |  |  |  |  | -0.01 (-0.04, 0.02) | 0.65 |
|  | MR-Weighted median |  | 2.00 | (1.16, 3.43) | 0.01 |  |  |  |  |  |  |

LDL cholesterol: low-density lipoprotein cholesterol; HDL cholesterol: high-density lipoprotein cholesterol; TC: total cholesterol; IVW: inverse variance weighted

Standard deviations (SDs) from GLGC (LDL cholesterol: 38.7 mg/dL; HDL cholesterol: 15.5 mg/dL; triglycerides: 90.7 mg/dL; TC: 41.8 mg/dL)
